# The Role of Free Radicals in Autophagy Regulation: Implications for Ageing

**DOI:** 10.1155/2018/2450748

**Published:** 2018-02-26

**Authors:** M. Pajares, A. Cuadrado, N. Engedal, Z. Jirsova, M. Cahova

**Affiliations:** ^1^Instituto de Investigaciones Biomédicas “Alberto Sols” UAM-CSIC, Instituto de Investigación Sanitaria La Paz (IdiPaz) and Department of Biochemistry, Faculty of Medicine, Autonomous University of Madrid, Madrid, Spain; ^2^Centro de Investigación Biomédica en Red sobre Enfermedades Neurodegenerativas (CIBERNED), ISCIII, Madrid, Spain; ^3^Centre for Molecular Medicine Norway (NCMM), Nordic EMBL Partnership for Molecular Medicine, University of Oslo, 0318 Oslo, Norway; ^4^Centre for Experimental Medicine, Institute for Clinical and Experimental Medicine, Videnska 1958 Prague, Czech Republic

## Abstract

Reactive oxygen and nitrogen species (ROS and RNS, resp.) have been traditionally perceived solely as detrimental, leading to oxidative damage of biological macromolecules and organelles, cellular demise, and ageing. However, recent data suggest that ROS/RNS also plays an integral role in intracellular signalling and redox homeostasis (redoxtasis), which are necessary for the maintenance of cellular functions. There is a complex relationship between cellular ROS/RNS content and autophagy, which represents one of the major quality control systems in the cell. In this review, we focus on redox signalling and autophagy regulation with a special interest on ageing-associated changes. In the last section, we describe the role of autophagy and redox signalling in the context of Alzheimer's disease as an example of a prevalent age-related disorder.

## 1. Introduction

In parallel with the increase in mean human life span over the recent decades, interest has grown in better understanding the underlying mechanisms of ageing and their roles in pathological conditions with a view to extending health span. Ageing hallmarks include genomic instability, telomere attrition, epigenetic alterations, deregulated nutrient-sensing, cellular senescence, stem cell exhaustion, altered intercellular communication, mitochondrial dysfunction, and loss of proteostasis [1]. Notably, several of these hallmarks may be related to progressive alterations in oxidative metabolism and accumulation of oxidatively damaged proteins, lipids, and nucleic acids during ageing [2]. However, this relationship is more complex than originally believed, since it has become increasingly clear that reactive oxygen species (ROS) and reactive nitrogen species (RNS) are not only detrimental to cells but can also have important roles as signalling molecules and participate in cellular functions such as cell-to-cell communication, proliferation, and survival in response to physiological cues and stress conditions [3].

Cellular functionality significantly depends on continuous maintenance and renewal of the whole proteome, that is, proteostasis (the loss of which defines one of the hallmarks of ageing). Two main cellular degradation systems are responsible for these functions: the ubiquitin-proteasomal system, which degrades individual proteins, and the autophagy-lysosomal system, which degrades whole organelles, protein aggregates, and long-lived proteins [4]. A prominent cause leading to the dysfunctionality of proteins is nonreversible oxidative modification. Proteolytic systems recognise and degrade such damaged proteins in order to prevent their accumulation and aggregation, thus preserving cellular viability. The general age-dependent decline in both proteasomal activity and autophagy, paralleled by accumulation of nondegraded dysfunctional material, has been reported in various mammalian models [5–9]. The underlying reasons for this decline are still a matter of debate but they include changes in the composition/structure of the degradation systems themselves [10, 11], increased accumulation of the material designated for degradation, resulting in exhaustion of the degradation systems [12, 13], or a combination of both.

In this review, we summarise the most relevant findings that describe the age-related dysregulation of autophagy in the context of redox alterations. We also provide evidence of how the dysregulation of autophagy and redox homeostasis (redoxtasis) with age is closely related to the development of one of the most prominent age-related diseases of our time, Alzheimer's disease.

## 2. The Dual Role of Free Radicals

### 2.1. ROS/RNS as Signalling Molecules

ROS/RNS are produced during cellular metabolism, or in response to xenobiotics, cytokines, and bacterial invasion, and can be generated in mitochondria or other cellular structures (e.g., peroxisomes, endoplasmic reticulum, and phagosomes) by a variety of enzymatic reactions. Single-electron transfer to oxygen along the mitochondrial electron transport chain (mETC) leads to a small fraction of partially reduced oxygen in the form of superoxide anion, O_2_^•−^ [14]. The prominent sites of superoxide formation are complex I (NADH dehydrogenase) and complex III (cytochrome bc1 complex). Other sources of O_2_^•−^ production include the NADPH oxidase family which are multicomponent enzymes specialized in the production for O_2_^•−^ in response to cellular stimuli involved in defense against pathogens or in cell proliferation [15]. Most other radical (hydroxyl radical OH^•^, NO^•^) and nonradical (H_2_O_2_) oxidative agents are derived from O_2_^•−^. Furthermore, O_2_^•−^ rapidly reacts with NO^•^ to form peroxynitrite (ONOO^−^) in high yields [16]. In addition, H_2_O_2_ is produced as a by-product of fatty acid and amino acid oxidation in peroxisomes [17] and by the oxidation of protein dithiols by the thiol oxidase ERO1 in the endoplasmic reticulum [18]. Beyond this, nonenzymatic sources of radicals such as the Fenton reaction have also been documented. Metal ions in reduced oxidation states (Fe^2+^, Cu^+^) can induce the catalytic decomposition of hydrogen peroxide and the concomitant formation of hydroxide anions (OH^−^) or extremely reactive hydroxyl radicals (OH^•−^). Their oxidized forms, Fe^3+^ and Cu^2+^, can be reduced by various electron donors (including superoxides) to restore the redox-active state [19].

ROS/RNS are important second messengers in a number of signal transduction pathways critical for cell growth and proliferation [20]. ROS/RNS influence the activity of key cellular enzymes (tyrosine kinases, serine-threonine kinases, and protein phosphatases) by reversible oxidation of sensitive amino acids (cysteine and methionine) located in their catalytic domains [21–23]. Well-documented targets of ROS signalling are protein phosphatases that contain a redox-sensitive cysteine residue in their catalytic center, for instance, protein tyrosine phosphatase 1B (PTP1B), a negative regulator of the insulin-signalling cascade. The cysteine of the catalytic center can be oxidised to sulphenic acid, leading to transient PTB1B deactivation and further to a sulphenyl-amide intermediate, which may prevent irreversible oxidation and facilitate PTP1B reactivation [24]. ROS/RNS also regulate the transcription of many crucial genes via the modification of key regulators of NRF2, NF*κ*B, HIF-1, and p53 transcription factors [25–28].

Another essential physiological function of ROS/RNS is the activation of the NLRP3 inflammasome upon infection by different pathogens [29–31]. Finally, ROS/RNS are critical mediators of cell death pathways, such as necrosis, apoptosis, and autophagy-programmed cell death [32]. The signalling function of ROS is facilitated by the existence of prominent redox sensors (mainly cysteines) within redox-regulated proteins known as “redox switches.” As they are prone to transient oxidation, ROS can transiently change the activity or localisation of redox switch-containing proteins [4].

NO, a second messenger that can impact on several molecular targets, is prone to oxidation by superoxide. The highly reactive product, peroxynitrite (ONOO^−^), can cause severe oxidative damage to biomolecules but can also potently modulate intracellular signalling by promoting the formation of 3-nitrosyl adducts with tyrosine moieties (“nitration”), as well as by less severe oxidation events. It is likely that nitration and oxidation of intracellular proteins by peroxynitrite are selective [33]. For instance, peroxynitrite can target receptor tyrosine kinase-signalling pathways [34]. Several mechanisms can mediate the peroxynitrite effects on tyrosine phosphorylation: (i) allosteric regulation of kinase activity by nitration or oxidation, (ii) modification of substrates, and (iii) peroxynitrite-induced modification (i.e., covalent dimerisation) of the receptors that subsequently attenuate kinase activity [35]. Regulation of receptor tyrosine kinases can occur due to modification of tyrosine phosphatase activity, as the active sites of many phosphatases contain cysteine thiolate, which normally serves as a transient acceptor of phosphate moieties but is inactive when oxidized by ROS/RNS. A typical example is PTP1B (previously referred to), which can be inactivated either by hydrogen peroxide or peroxynitrite. While H_2_O_2_ oxidation results in reversible cysteine oxidation to sulphenic acid, peroxynitrite catalyses irreversible sulphinic and sulphonic acid formation and terminal inactivation of the enzyme [35].

### 2.2. Redox Homeostasis (Redoxtasis)

“Oxidative stress” was first formulated as a biological concept in 1985 [36] and has since enormously affected many areas of biological research. However, the meaning of the term has substantially changed over the years. The concept of oxidative stress was initially perceived as a simple imbalance between the formation of free radicals and their elimination by antioxidant defense systems. During subsequent decades, it became more and more apparent that free radicals are utilised as signals or regulators in many fundamental cellular processes. Thus, the concept of oxidative stress has now been updated to include the role of redox signalling and redefined as “a disturbance in the prooxidant-antioxidant balance in favour of the former leading to the disruption of redox signalling” [37].

Since proteins are the largest group of macromolecules, they are the most frequent targets of ROS/RNS. Thus, levels of protein carbonyls or nitrotyrosine are used as biomarkers of oxidative stress [2]. Protein oxidation gradually results in the loss of activity, unfolding and exposure of hydrophobic patches, facilitating aggregation, and cross-linking and eventually rendering proteins resistant to proteolysis. Moreover, oxidative stress is closely related to the presence of advanced glycation end products (AGEs)—as a result of the chemical reaction between proteins and reducing carbohydrates—and to advanced lipid peroxidation end products (ALEs)—derived from the reaction between proteins and lipid peroxidation products. AGEs and ALEs represent a very heterogeneous class of molecules, which are formed by different pathways either exogenously (in food or tobacco smoke) or endogenously [38]. AGE- and ALE-modified proteins are characterised by the loss of structural and functional properties. For instance, glycated extracellular matrix proteins can inhibit the cell adhesion and migration of T-cells, accompanied by decreased actin polymerisation [39]. In addition to their direct, toxic effects, AGEs and ALEs can influence cell surface receptors. For example, the receptor for AGEs (RAGE) is expressed on the surface of various cell types [40] and mediates the induction of ERK and p38-MAPK signalling cascades [41, 42] as well as the activation of NADPH oxidase, enhancing ROS generation [43].

### 2.3. Cellular Mechanisms for Maintaining Redoxtasis

To protect themselves from excessive oxidative stress, organisms have developed a number of different response systems designed to sense and rapidly respond to changing levels of specific oxidants [44]. These mechanisms include (i) the endogenous antioxidant systems, (ii) transcriptional changes mediated by oxidative modification of specific transcription factors [45], (iii) activation of specific chaperones which protect against oxidative protein aggregation [46, 47], (iv) metabolism redirection (from energy production towards NADPH generation) by altering the activity of key enzymes involved in energy metabolism [48], and (v) activation of specific degradation systems (proteasomal degradation and/or autophagy) in order to eliminate damaged components.

#### 2.3.1. Endogenous Antioxidant Systems

Endogenous antioxidant systems include low-molecular antioxidants such as vitamins, glutathione (reduced GSH and oxidized GSSG), lipophilic antioxidants, and uric acid, among others. Moreover, the electron donor groups, peroxiredoxins (PRXs), thioredoxins (TRXs), and glutaredoxins (GRXs) are considered guards of the intracellular redox state and key regulators of redox signalling.

Peroxiredoxins (PRXs) reduce hydrogen peroxides, organic hydrogen peroxides, and peroxynitrites [49]. They also translate information about the increased intracellular levels of oxidants into effector systems through the modification of signalling cascades. The catalytic active sites of PRXs and other thiol peroxidases contain cysteine, which is prone to oxidation by H_2_O_2_ and rapidly undergoes sulphenic acid formation [50]. Subsequently, this sulphenic acid reacts with thiol groups in target proteins, resulting in oxidation and regeneration, that is, the reduction of thiol peroxidase. The high redundancy of thiol peroxidases indicates their importance in cellular stress adaptations. Under conditions of high oxidative stress, sulphenic acid is further oxidised to sulphinic acid. Although “overoxidised” peroxiredoxins lose their antioxidant functions, they switch to molecular chaperones that expose their hydrophobic surfaces in order to bind protein-folding intermediates and prevent protein aggregation. The sulphinic acid in peroxiredoxins can only be reduced by mitochondrial sulphiredoxins [51, 52]. Thiol oxidation products can be reduced by thioredoxins (TRXs) or glutaredoxins (GRXs) [53]. TRXs prefer sulphenic acids, while GRXs can catalyse both S-glutathionylation and deglutathionylation, depending on the relative concentrations of GSH and GSSG. Under conditions where the GSH/GSSG ratio is decreased, that is, under the action of oxidising factors, GRXs can catalyse the S-glutathionylation reaction, while under weakening oxidative stress, GRXs can catalyse deglutathionylation [54, 55]. In contrast to the above-described redox switches, redox sensing in GRXs is dependent not only on reactive cysteines but also on Fe/S clusters stabilised by glutathione, which is derived from free-GSH pools [56–58]. GRXs may influence intracellular redox signalling by S-glutathionylation of effector proteins with different outcomes. S-glutathionylation catalysed by glutaredoxin has been shown to inhibit phosphofructokinase, glyceraldehyde-3-phosphate dehydrogenase, and PTP1B, among others. In contrast, proteins such as microsomal S-glutathione transferase, HRAS GTPase, and complex II of the mitochondrial respiratory chain are activated by S-glutathionylation [59].

#### 2.3.2. Transcription-Dependent Control of Redoxtasis

An example of a redox-sensitive transcription factor is nuclear factor erythroid-derived-like 2 (NRF2). NRF2 activity is subject to a tight and multilevel control. The redox sensor KEAP1 enables NRF2 levels to adjust to oxidant fluctuations. Under basal conditions, NRF2 is sequestered by a KEAP1 homodimer, an E3-ligase that presents NRF2 to the CULLIN3/RBX1 protein complex, resulting in ubiquitination and proteasomal degradation. However, KEAP1 contains several key cysteines that can be oxidised, resulting in a conformational change that prevents the presentation of NRF2 to the proteasomal machinery and thus enabling newly synthesised NRF2 to accumulate and activate the expression of antioxidant response element- (ARE-) controlled genes. Several antioxidant and detoxifying enzymes as well as anti-inflammatory and proteostatic mediators are coded by NRF2-target genes [25, 60].

#### 2.3.3. Chaperones

Oxidised proteins can lose their structure and become prone to aggregate. Thus, it is not surprising that specific chaperones are activated under oxidative stress conditions. The heat shock protein Hsp33 is normally inactive because of a highly conserved cysteine-containing zinc center. Oxidation leads to the formation of two intramolecular disulphide bonds accompanied by zinc release, which facilitates the formation of active, oxidised Hsp33 dimers. These dimers bind tightly to substrate proteins and prevent irreversible aggregation. Once redoxtasis is recovered, Hsp33 is reduced and the substrate protein is released [46, 47].

#### 2.3.4. Degradation Systems

If redox imbalance exceeds the cellular antioxidant capacity, macromolecules and even organelles can suffer from oxidative damage. Fortunately, mammalian cells rely on a complex network of degradation systems, which guarantees the elimination of altered intracellular components such as oxidised proteins. The role of the ubiquitin-proteasome system (UPS) in the degradation of oxyproteins has been extensively addressed [4, 61–63]. In this review, we will focus on the autophagy process, which degrades not only soluble proteins but also aggregates and even organelles.

### 2.4. Autophagy and the Maintenance of Proteostasis

In addition to its importance in cellular recycling and energy supply during starvation, autophagy is now recognised as a critical housekeeping pathway in a broader range of conditions, including oxidative stress. The term “autophagy” encompasses all the processes by which cellular components (proteins, organelles, aggregates, and intracellular pathogens) are supplied to lysosomes for degradation. Different types of autophagy coexist in mammals, depending on the way in which cargoes are delivered to lysosomes. These mechanisms comprise macroautophagy, chaperone-mediated autophagy, and microautophagy.

#### 2.4.1. Macroautophagy

Macroautophagy (often referred to as autophagy) is a process whereby portions of the cytoplasm are sequestered by the expansion and closure of compressed membranous cisterna (termed “phagophores”), to produce double- or multiple-membraned vesicles called “autophagosomes,” which eventually fuse with lysosomes for degradation of the inner autophagosomal membrane and the sequestered content. Yeast studies have identified more than 30 autophagy-related proteins (ATGs) that are important for the autophagic process, and many of their orthologues have also been identified in mammals. The macroautophagic process in mammals is extremely complex, as different regulatory mechanisms can operate in distinct cell types and under different conditions in order to maintain proteostasis. Briefly, the ULK complex (formed by ULK1/ULK2-ATG13-FIP200-ATG10l) is activated by different signals, such as the energy sensor AMPK, enabling phagophore nucleation and assembly. The activated ULK complex targets and recruits a class III phosphatidylinositol-3 kinase complex (PI3K/VPS34-BECLIN1-VPS15-ATG14) to locally produce phosphatidylinositol-3-phosphate in the phagophore membrane, which serves to recruit other proteins to the nucleation site. The phagophore expansion step is associated with two ubiquitination-like reactions. First, ATG7 acts as an E1 ubiquitin-activating enzyme and ATG10 as an E2 ubiquitin-conjugating enzyme, enabling ATG12 conjugation to ATG5. Second, ATG12-ATG5 complexes interact noncovalently with ATG16L. This complex acts as an E3-ligase, facilitating the second ubiquitin-like reaction, where LC3 and GABARAP proteins are conjugated to phosphatidylethanolamine (PE) by ATG7 (E1-like) and ATG3 (E2-like) to form LC3-II and GABARAPs-II anchored to the phagophore membrane. Different autophagy cargo receptors, such as p62/SQSTM1 and NDP52, interact with ubiquitin-containing proteins as well as with LC3s and GABARAPs, enabling specific substrates to be engulfed by autophagosomes and delivered, through dynein-dependent movement along microtubules, to lysosomes, where fusion subsequently occurs (mediated by SNARE proteins). ATG12-ATG5 complexes dissociate from the autophagosomal membrane once autophagosome formation is complete, while ATG4 is necessary for the delipidation and recycling of LC3-II and GABARAPs-II, as well as for the initial proteolytic activation of newly expressed pro-LC3 and pro-GABARAP proteins. The resulting breakdown products inside lysosomes are released through permeases for recycling in the cytosol [64].

#### 2.4.2. Chaperone-Mediated Autophagy (CMA)

Chaperone-mediated autophagy (CMA) is a type of autophagy that facilitates the selective degradation of soluble proteins containing a specific KFERQ-like motif. The chaperone HSC70 recognises and binds to proteins bearing this pentapeptide [65]. HSC70 delivers substrate proteins to lysosomes, where they interact with the lysosomal receptor LAMP2A [66]. LAMP2A then multimerises and generates a translocon, which enables the susbstrate protein to enter the lysosome, assisted by lysosomal HSC70 (lysHSC70) and other sets of chaperones/cochaperones [67, 68].

#### 2.4.3. Microautophagy

Microautophagy is the third type of autophagy in mammals and the least studied thus far. It involves the direct invagination of the lysosomal membrane, resulting in the engulfment of cytosolic cargoes that are then degraded by lysosomal proteases [69].

## 3. Redox Signalling and Autophagy Regulation

Many reports have demonstrated that redox signalling affects autophagic flux, generally resulting in its induction (reviewed in [61]). This may represent a cell survival mechanism, as autophagy enables the removal of damaged structures (protein and organelle homeostasis) and provides surplus energy substrates. For instance, upregulation of autophagy by rapamycin, lithium, carbamazepine, and valproic acid in SHSY5Y cells has been shown to protect against rotenone (a natural ROS-generating compound) toxicity in an ATG5-dependent manner [70, 71].

However, prolonged autophagy may result in the degradation of essential proteins and organelles and cell death (autophagic cell death or programmed cell death type II, PCD II) [72]. For example, increased SOD2-mediated H_2_O_2_ formation has been shown to facilitate autophagic, Atg5-dependent cell death in senescent keratinocytes through the accumulation of autophagic markers [73]. The outcome of autophagy induction is thus context-dependent, as it depends on the level, localisation, and type of ROS/RNS.

**Figure 1:**
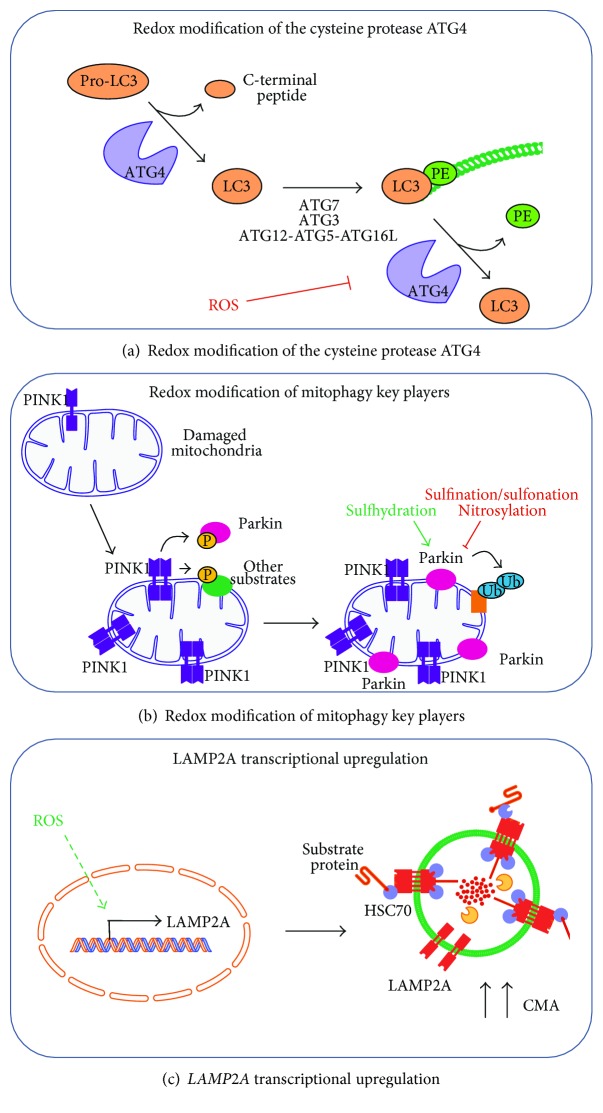
Redox modification of autophagy core components. (a) Cysteine protease ATG4 is sensitive to redox modification. ATG4 cleaves the C-terminal peptide in LC3 (or GABARAPs), making it a suitable substrate for conjugation to phosphatidylethanolamine (PE), which is mediated by ATG7, ATG3, and the ATG12-ATG5-ATG16L complex. LC3 conjugated to PE (LC3-II) is inserted into the autophagosomal membrane and enables it to elongate. ATG4 also acts as a delipidating enzyme, releasing LC3 from PE. ROS are essential for regulating ATG4 activity, as redox modification of cysteine residues transiently inhibits delipidation activity in order to promote autophagosome formation. (b) Mitophagy core components are targets of redox modification. Briefly, damaged mitochondria result in the stabilisation, dimerisation, and activation of kinase PINK1 in the organelle. PINK1 phosphorylates Parkin and other substrates, which further recruit Parkin to the mitochondrial membrane. Parkin acts as an E3-ubiquitin ligase, ubiquitinating several substrates that are recognised by autophagy receptors in order to direct mitochondria toward lysosomal degradation. Physiological sulfhydration enables, whereas pathological nitrosylation or sulphination/sulfonation inhibits, Parkin catalytic activity. (c) Mild oxidative stress upregulates chaperone-mediated autophagy (CMA) by transcriptional induction of lysosomal receptor LAMP2A.

**Figure 2:**
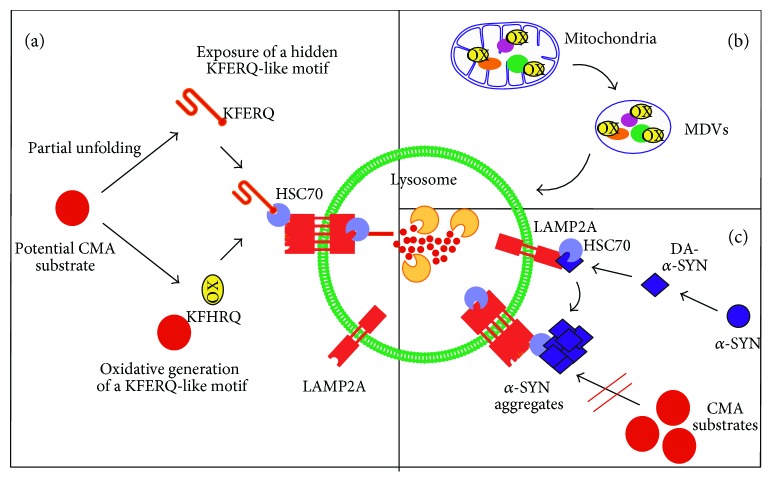
Redox modification of autophagy targets. (a) Oxidative-modified targets are better substrates for CMA degradation. Possible explanations for the increased degradation of oxidised substrates by CMA include (i) partial unfolding of substrates facilitating lysosomal translocation; (ii) partial unfolding of substrates exposing hidden KFERQ-like motifs; (iii) generation of a new KFERQ-like motif due to specific oxidation of amino acid residues. (b) The enrichment of oxidised substrates in mitochondrial-derived vesicles (MDVs) points to a mitochondrial quality control mechanism under oxidative stress conditions. (c) Specific redox modification of targets involved in disease. The interaction of oxidised dopamine with *α*-synuclein (*α*-SYN) generates dopamine-modified *α*-SYN (DA-*α-*SYN), which is poorly degraded by CMA; it instead forms oligomers and aggregates, further blocking the degradation of other CMA substrates.

As the first line of defense against oxidative stress, posttranslational modification of key proteins of the autophagic-lysosomal pathway leads to an instant increase in autophagic flux. When oxidative stress becomes chronic, a long-term response can be generated via the activation of specific transcriptional networks (NRF2, NF*κ*B, p53, and FOXO3). In addition to their effect on autophagy machinery proteins (Figure 1), oxidants can also modify autophagy targets, thereby increasing or decreasing their susceptibility to degradation (Figure 2).

### 3.1. Redox Modification of Key Upstream Autophagy Regulators

#### 3.1.1. 5′AMP-Activated Protein Kinase (AMPK)

5′AMP-activated protein kinase (AMPK) senses cellular stress and triggers the activation of several prosurvival pathways, including autophagy. AMPK may stimulate autophagy either indirectly via mTOR inhibition or directly via phosphorylation of ULK1 [74–76]. ROS and RNS can oxidise cysteine residues in both the *α*- and *β*-AMPK subunits, generating S-glutathionylated derivatives with increased kinase activity [77, 78]. Additionally, intracellular ROS can trigger Ca^2+^ release from the endoplasmic reticulum, while subsequent CaMKK*β* activation also results in AMPK activation [79]. AMPK may be activated by hypoxia via ROS generated within the mitochondrial electron transport chain [80]. Moreover, starvation-induced ROS have been shown to induce AMPK-dependent autophagy, while cells overexpressing the antioxidant enzyme manganese-superoxide dismutase 2 (SOD2) fail to activate AMPK following starvation [81].

#### 3.1.2. Ataxia Telangiectasia Mutated Protein Kinase (ATM)

Ataxia telangiectasia mutated protein kinase (ATM) is a tumour suppressor protein crucial to the DNA damage-repair response. ATM exists in two different cellular pools: (i) nuclear ATM, involved in DNA repair and (ii) cytoplasmic ATM, which acts as a ROS sensor and an activator of the tuberous sclerosis complex 2 (TSC2) tumour suppressor by signalling to LKB1 and AMPK to relieve mTORC1 repression of autophagy [82]. The exact mechanisms employed by ATM in order to sense increased ROS concentration are yet unclear, but two possible mechanisms have been proposed. On the one hand, ATM contains many cysteine residues that are potential targets for direct oxidation by ROS. On the other, the signal may be mediated by oxidised lipid intermediates, since ATM colocalises with different membrane compartments in the cell.

#### 3.1.3. Mitogen-Activated Protein Kinases (MAPK)

Mitogen-activated protein kinases (MAPK) can be activated by ROS signalling, in a manner that can impact autophagy outcome. For instance, the kinase ASK1 binds to a reduced form of thioredoxin (TRX), which prevents dimerisation and activation. Oxidative stress promotes oxidation and dissociation of TRX and autophosphorylation of ASK1 and upregulation of its kinase activity [83]. ASK1 can phosphorylate and activate c-Jun N-terminal kinase 1 (JNK1). In turn, JNK1 can phosphorylate BCL-2 at multiple sites, thus disrupting its inhibitory interaction with BECLIN1 and favouring phagophore nucleation [84]. On the other hand, inhibition of JNK1 by NO reduces BCL-2 phosphorylation and increases the BCL-2-BECLIN1 interaction, thus inhibiting autophagy. It has also been observed that BCL-2 can be directly S-nitrosylated, which inhibits degradation and stabilises protein levels [85, 86].

ROS/RNS can also lead to sustained activation of the extracellular signal-regulated kinase (ERK) pathway, by either direct oxidation or nitration of the upstream activators RAF and MEK or inhibition of dual-specificity protein phosphatases or PP1/2A. ERK leads to phosphorylation and inactivation of TSC2, impairing its ability to inhibit mTOR signalling and thus suppresses autophagy [87].

#### 3.1.4. KEAP1/NRF2

As previously mentioned, reactive cysteines in KEAP1 act as redox sensors, which, when oxidised, generate a conformational change in KEAP1 that renders it incapable of presenting NRF2 to the proteasomal machinery. As a consequence, NRF2 accumulates, translocates to the nucleus, and induces the expression of its target genes. The first direct link between NRF2 and autophagy was reported in connection with the autophagy receptor protein p62, which competes with NRF2 in binding to KEAP1 [88–90]. It has been suggested that the binding of p62 to KEAP1 leads to autophagic degradation of KEAP1, since silencing of p62 doubles the KEAP1 half-life [91, 92]. Phosphorylation of p62 increases its binding affinity to KEAP1, facilitating NRF2 accumulation and transcriptional activation of its target genes [88, 93]. It was also shown that TGF-*β*-activated kinase 1 (TAK1) can phosphorylate p62, enhancing KEAP1 degradation and NRF2 upregulation. TAK1 deficiency upregulates ROS in the absence of any exogenous oxidant in parallel with a reduction in NRF2 protein levels suggesting that TAK1/p62/NRF2 axis is a way to regulate cellular redoxtasis under steady-state conditions [94].

NRF2, in turn, regulates the expression of relevant genes for macroautophagy, including ULK1, p62, NDP52, ATG4D, ATG7, GABARAPL1, ATG2B, and ATG5 [93, 95–97]. Therefore, it seems that NRF2 activation increases macroautophagy, which in turn results in KEAP1 degradation and favours further NRF2 stabilization in a positive feedback loop. This mechanism of NRF2 induction might be a relevant response to prolonged cellular stress.

#### 3.1.5. IKK/NF*κ*B

Nuclear factor kappa-light-chain-enhancer of activated B-cell (NF*κ*B) signalling and autophagy is reciprocally involved in the control of cellular survival under conditions of stress. In an unstimulated state, NF*κ*B renders in the cytosol as an inactive complex with I*κ*B. Various conditions of stress, including oxidative stress, activate upstream I*κ*B kinases (IKK) that phosphorylate I*κ*B, leading to its ubiquitination and proteasomal degradation. Autophagy induction by nutrient starvation or mTOR inhibition by rapamycin correlates with IKK activation and I*κ*B degradation but not necessarily with the activation of NF*κ*B [98]. Constitutive activation of the IKK complex involved hyperphosphorylation-dependent activation of AMPK, hypophosphorylation of the mTOR substrate p70^S6K^, depletion of p53 protein, and release of BECLIN1 from the inhibitory complex with BCL-2. Autophagy induction by constitutively active IKK could be prevented by knockdown of the *α*-subunit of AMPK suggesting that IKK-stimulated autophagy is controlled by the canonical AMPK/mTOR pathway [98]. These data indicate that the IKK complex may induce autophagy, but experiments with mouse embryonic fibroblasts knocked out for IKK subunits revealed that IKK is not indispensable [99]. More likely, IKK is required for the optimization of autophagy induced by physiological and pharmacological stimuli. During onset of stress conditions, IKK stimulates autophagy via NF*κ*B-independent increased expression of ATG5, BECN1, and LC3 [100], but IKK itself can be inactivated by S-nitrosylation [85]. Conversely, autophagy may contribute to the regulation of the IKK pathway as all three IKK subunits (*α*, *β*, and *γ*) as well as their upstream activator NF*κ*B-inducing kinase are degraded by the autophagic pathway [101].

Released NF*κ*B can translocate to the nucleus and function as an efficient transcription factor [102]. The role of NF*κ*B in autophagy regulation is ambiguous. On the one hand, it can promote autophagy by transactivating the proautophagy protein BECLIN1 [103]. On the other hand, TNF*α* was shown to repress autophagy via NF*κ*B-dependent activation of the autophagy inhibitor mTOR in Ewing sarcoma cells. In cells lacking NF*κ*B, TNF*α* treatment upregulated the expression of BECLIN 1 and subsequently induced an accumulation of autophagic vacuoles. Both of these responses were dependent on ROS production [104]. Interestingly, Atg5- and Atg7-deficient mouse embryo fibroblasts are unable to activate the NF*κ*B pathway in response to TNF*α*, which points to a role of autophagy in NF*κ*B activation [105].

NRF2- and NF*κ*B-signalling pathways must be well coordinated in order to keep the fragile balance between the antioxidative and proinflammatory processes that occur in the cell. One of the underlying mechanisms of crosstalk depends on autophagy. Free KEAP1 can prevent IKK from binding to heat shock protein 90 (Hsp90) [106] thereby inducing autophagic degradation of IKK and attenuating NF*κ*B signalling [107].

#### 3.1.6. Sirtuin 1

Sirtuin 1 (SIRT1), a class III histone deacetylase, is a key component of the cellular prosurvival pathway during the response to stress conditions [108]. The excessive presence of ROS induces SIRT1 activation and translocation to the nucleus via two independent effector pathways, namely, JNK1 and AMPK [108]. Nevertheless, SIRT1 itself can be a target of oxidative modification specific of cysteine residues, which enhance its degradation by the proteasome [109]. Activated SIRT1 is a potent inducer of autophagy and exerts its effect either directly via interaction with components of the autophagy cascade or indirectly via FoxO signalling. SIRT1 can participate in a molecular complex with several essential components of the autophagy machinery, including ATG5, ATG7, and LC3. It can also deacetylate them, thus promoting autophagosome formation [110]. FoxO proteins belong to a family of transcription factors that are activated during conditions of cellular stress. SIRT1 deacetylates FoxO1, which subsequently stimulates expression of RAB7, a protein essential for autophagosome fusion with lysosomes [111]. FoxO3, another member of the FoxO family, can also be deacetylated by SIRT1 in response to oxidative stress, stimulating the expression of the autophagy proteins LC3 and BNIP3 in skeletal muscle [112].

### 3.2. Redox Modification of Autophagy Core Proteins

#### 3.2.1. Autophagy-Related Protein 4 (ATG4)

ATG4 is an important member of the autophagy cascade, and it is essential for autophagosome formation. It has the dual role of, first, cleaving LC3 and GABARAPs at the C-terminus so that they can be conjugated to phosphatidylethanolamine (PE) and, second, cleaving (deconjugating) LC3 and GABARAPs from the already-formed autophagosomal membrane. ATG4 contains reactive cysteines prone to oxidation by ROS (specifically by H_2_O_2_, generated upon starvation), which reversibly inhibit ATG4 activity [113]. It was proposed that starvation induces a local production of H_2_O_2_ in the vicinity of the autophagosome formation site [113]. This would locally inactivate ATG4, so that it cannot deconjugate PE-conjugated LC3 and GABARAPs on the phagophore, thus allowing autophagosome formation. As autophagosomes are trafficked towards lysosomes, they presumably arrive to environments with lower H_2_O_2_ concentrations, allowing ATG4 reactivation, and thus the deconjugation and recycling of LC3 and GABARAPs (Figure 1(a)).

#### 3.2.2. Mitophagy Players

Mitophagy is a specific type of autophagy in which mitochondria are targeted for lysosomal degradation. The E3-ubiquitin ligase Parkin translocates to damaged mitochondria and is one of the key regulators of mitophagy induction [114]. Parkin contains two highly conserved cysteines. Their mutation has been linked to Parkinson's disease and results in the loss of Parkin activity and impaired mitophagy [115], which points to the importance of these redox-sensitive residues. A study by Meng et al. showed that sulphination/sulfonation of key cysteine residues in Parkin, as well as in protein regions affected by familial mutations, led to decreased activity of the enzyme and contributed to protein aggregation [116]. Moreover, Vandiver et al. reported that Parkin is physiologically sulfhydrated and that, whereas nitrosylation inactivates it, sulfhydration stimulates its catalytic activity [117] (Figure 1(b)). Another protein implicated both in the antioxidant response and in mitochondrial removal is DJ-1. Similar to what occurs with Parkin, DJ-1 is susceptible to redox signalling. Thus, oxidation of a specific cysteine in this protein is necessary for mitochondrial targeting and protection against oxidation-induced cell death [118].

#### 3.2.3. LAMP2A

Although there have been no reports of a direct oxidative modification of the lysosomal receptor for CMA (LAMP2A), mild oxidative stress leads to increased LAMP2A levels, together with augmented lys-HSC70 and the cochaperones HIP and HSP90. In contrast to other CMA-activating stimuli, such as nutrient deprivation, increased LAMP2A levels under oxidative stress are achieved transcriptionally [119] (Figure 1(c)).

### 3.3. Redox Modification of Autophagy Targets

#### 3.3.1. CMA Targets

CMA is required for preserving cell viability in response to oxidative stress. Thus, exposure of CMA-incompetent cells to oxidant and prooxidant factors (H_2_O_2_, paraquat, and cadmium) results in more severely compromised cell viability than in cells with preserved CMA function [120]. In fact, increased levels of oxidised proteins can be found in the lysosomal lumen under mild oxidative stress conditions, presumably due to the higher binding and uptake of substrates [119]. Incubation of well-known CMA substrates and a pool of cytosolic proteins with increasing amounts of prooxidants accelerate degradation by CMA. The mechanisms by which ROS facilitates degradation have not been fully elucidated. One possible explanation is that protein oxidation causes partial unfolding, not only exposing hidden recognition motifs to HSC70 but also facilitating translocation to the lysosomal lumen (Figure 2(a)). Another possibility is that oxidation of certain residues creates a previously nonexisting KFERQ-like motif. For instance, positive histidine when oxidized will resemble a negative aspartic acid residue [121].

#### 3.3.2. Mitochondrial-Derived Vesicles (MDVs)

A new mechanism for maintaining mitochondrial quality control, different from mitophagy, has recently been described [122]. Mitochondrial-derived vesicles (MDVs) are generated by a budding process from mitochondria in order to selectively transport mitochondrial proteins to either the peroxisomes or the lysosomes for degradation. MDVs are stimulated under different stress conditions and contain specific cargoes depending on the nature of the insult. Interestingly, Soubannier et al. showed enrichment in oxidised cargoes within these vesicles [123]. This process may represent a quicker mitochondrial quality control mechanism than mitophagy, as it preserves mitochondrial function by selectively degrading damaged mitochondrial proteins (Figure 2(b)).

#### 3.3.3. Specific Proteins Involved in Disease

Specific individual proteins have been extensively analysed for oxidative modifications because of their involvement in disease. One example is *α*-SYN due to its aberrant accumulation in Parkinson's disease. Oxidation and nitration of *α*-SYN stabilises protein polymers by forming stable cross-linked *α*-SYN aggregates. Using HEK293 cells stably transfected with wild-type and mutant *α*-SYN, Paxinou et al. demonstrated that intracellular generation of nitrating agents results in the formation of *α*-SYN aggregates and prevents them from being degraded in lysosomes [124]. Dopaminergic neurons are thought to be particularly vulnerable to nitrosative/oxidative damage. Interestingly, a modified form of *α*-SYN, resulting from a noncovalent interaction with oxidised dopamine, has been suggested to be responsible for neuron toxicity [40]. While *α*-SYN is, at least in part, degraded by CMA [125], mutant and dopamine-modified forms of *α*-SYN are no longer properly degraded by this pathway. Instead, these forms of *α*-SYN tend to aggregate and prevent degradation of other substrates, further impairing proteostasis and increasing susceptibility to oxidative stress [125, 126] (Figure 2(c)). Other examples will be extensively analysed in the context of Alzheimer's disease (AD) in Section 5.

## 4. Changes in ROS Signalling and Autophagy with Ageing

Ageing is associated with the accumulation of oxidatively modified proteins. The final burden of dysfunctional proteins depends on multitude of factors that govern (a) the rates of formation of various kinds of ROS, (b) the levels of antioxidant defenses that guard against ROS-mediated protein damage, (c) the sensitivity of proteins to oxidative attack, and (d) the capacity of the cell to repair or eliminate damaged proteins [2].

### 4.1. Ageing and the Antioxidant Defense System

Although some studies support the premise that antioxidant enzyme function does not generally decrease with age [127], reduced capacity of specific antioxidant systems has been shown to develop with age [128]. One supporting example is the fact that levels of methionine sulphoxide increase with age in humans (i.e., in cataractous lenses, trabecular meshwork, skin collagen, or senescent erythrocytes), probably due to decreased methionine sulphoxide reductase (MSR) activities [129]. Interestingly, no increase in methionine sulphoxide was found in aged mouse tissues [129]. Studies performed in *D. melanogaster* or mice demonstrated that loss-of-function mutations in MSR correlates with reduced maximal life span, while MSR-overexpression results in extended life span [130–132]. This is also the case for transcription factor NRF2, the master regulator of the antioxidant cell response (reviewed by Bruns et al.) [133]. Reduced binding of NRF2 to its antioxidant response element (ARE) has been observed in aged rodents in parallel with reduced glutathione levels [134]. Studies in *D. melanogaster* have shown reduced NRF2/CncC responsiveness to stress. Overexpression of the NRF2/CncC partner Maf restored NRF2/CncC signalling competence and antagonised age-associated functional decline [135]. These and other studies support the hypothesis that the inability of the organism to adapt to internal and external conditions contributes to age-related loss of homeostasis [127, 135].

### 4.2. Macroautophagy Decline in Ageing

Plenty of evidence shows that a decline in the capacity of proteolytic systems occurs with age. In fact, the overall reduced rates of protein degradation with age were first observed almost three decades ago [136, 137].

The crucial role of autophagy in the ageing phenotype is reflected by studies in which loss-of-function mutations or deficient expression of several autophagy-related genes results in decreased life span in different organisms [8]. Matecic et al. performed an unbiased screen for ageing factors in the yeast *S. cerevisiae*, which led them to identify many short-lived mutants with autophagic defects [138]. Conversely, several reports demonstrate that the activation of macroautophagy (genetically, pharmacologically, or by calorie restriction) extends the life span of various organisms [139]. For example, brain-specific overexpression of Atg8 (the orthologue of LC3/GABARAPs) and treatment with spermidine has been shown to induce autophagy and extend life span in *D. melanogaster* flies [140, 141]. In mice, treatment with the autophagy activator rapamycin slows age-related alterations and prolongs longevity [142, 143]. Overexpression of ATG5 in mice resulted in extended life span along with antiageing phenotypes, including leanness, increased insulin sensitivity, and improved motor function. Interestingly, cultured fibroblasts from these mice were more resistant to oxidative damage in an autophagy-dependent manner [144]. In fact, it has been suggested that the long-lived naked mole rat copes with chronic oxidative stress by enhancing its proteostatic network [145].

Various studies have revealed a decrease with age in both the formation and subsequent elimination of autophagosomes in different tissues of aged animals [146, 147]. The levels of core autophagy proteins in various tissues from distinct aged organisms have been analysed. The expression of many components of the autophagy pathway is reported to be reduced with age in *Drosophila* muscles (i.e., Atg1, Atg5, Atg6, Atg7, and Atg8) [148]. LC3 and ATG7 levels have been shown to be downregulated in the muscles of aged mice and humans [149]. Another study reported downregulation of several autophagy-related genes (e.g., Atg5, Atg7, and BECN1) in the human aged brain [150]. Ott et al. reported reduced levels of ATG5-ATG12, LC3-II/LC3-I ratios, BECLIN1, and p62 in aged murine brain tissue and senescent human fibroblasts [151]. However, the precise mechanisms that lead to reduced expression of ATGs remain unclear. Dysregulation of signalling pathways that regulate autophagy may also contribute to the age-related decline in autophagy. For instance, the stimulatory effect of glucagon on macroautophagy is blunted with age, while the inhibitory effect of insulin remains intact [152].

#### 4.2.1. Lipofuscin Accumulation

Defective autophagy may favour the accumulation of lipofuscin with age. Oxidised proteins may not undergo adequate proteolytic digestion but instead cross-link with one another or form extensive hydrophobic bonds [153]. These cross-linked proteins can react with other cellular components and generate an autofluorescent material called “lipofuscin,” a nondegradable polymeric substance consisting of proteins (30–70%), lipids (20–50%), and sugar residues (7%) [154]. 99% of lipofuscin colocalises with lysosomes, whereas only 1% is found in the cytosol [4]. Although macroautophagy is responsible for the uptake of lipofuscin into lysosomes, experiments using an ATG5-knockout model showed that inhibition of macroautophagy does not prevent lipofuscin formation but rather leads to accumulation of cytosolic lipofuscin with enhanced cytotoxicity [155]. Lysosomes are the degradation site for iron-containing metalloproteins, such as cytochromes and ferritin, resulting in the release of redox-active low-molecular-mass iron. In ferrous form, ferritin reacts with hydrogen peroxide (which easily diffuses throughout the cell), forming the extremely reactive hydroxyl radical via the Fenton reaction. Hydroxyl radicals are highly unstable and react with fatty acids to form organic peroxides and aldehydes, which can react with one or two free amino groups within proteins, forming Schiff bases. The formation of aldehyde bridges, an important mechanism of protein-protein cross-linking, is involved in lipofuscinogenesis [156].

While mitotic cells are able to “dilute” lipofuscin via ongoing cell division, this pigment particularly accumulates in postmitotic tissues with age. In fact, lipofuscin can fill up to 40% of the cytosolic volume in aged animals. Lipofuscin-loaded lysosomes are no longer considered “residual bodies,” as they have been shown to receive new lysosomal enzymes in an attempt to degrade lipofuscin. Nonetheless, the accumulation of lipofuscin in lysosomes also impairs efficient lysosomal degradation of other substrates. Indeed, lipofuscin-loaded human fibroblasts exhibit reduced autophagy under conditions of starvation [157]. Moreover, lipofuscin inhibits proteasomal activity [158] and is considered a source of oxidative stress because of the incorporation of transition metals. Overall, lipofuscin impairs degradation of other proteins and increases the potential for further oxidative damage [159].

#### 4.2.2. Formation of Advanced Glycation Products

Several studies have confirmed the accumulation of AGEs and ALEs with age in different tissues, including rodent and human skin [136], eye lens [160], renal arteries [161], and intervertebral discs [161]. Many studies indicate that the amount of AGEs in certain tissues correlates with the half-lives of their proteins. For example, higher levels of AGEs have been found in cartilage collagen (half-life of 117 years) compared to skin collagen (half-life of 15 years) [162]. It would seem that intracellular proteins are protected from transformation to AGEs because of their fast turnover. However, the proteolytic capacity of the cell decreases with age, making proteins more susceptible to glycation. Furthermore, lysosomal proteases may be inhibited by glycating agents and AGE-modified proteins, which will limit degradation and allow accumulation of AGEs [163, 164].

#### 4.2.3. Transcriptional Regulation

Transcriptional regulation of autophagy may also be affected with age. As previously mentioned, one example is the reduction in NRF2 activity, which may in turn result in reduced expression of antioxidant enzymes as well as core components of proteostasis machineries [97]. Another transcription factor closely connected with redoxtasis, autophagy, and longevity is FoxO. A recent study reported an age-dependent decrease in the expression of FoxO and some of its target genes in the intervertebral discs of mice. This may also be the case in humans, where decreased FoxO levels have been found in degenerating discs [165]. Only further studies can determine whether this is the case for other tissues. Whether TFEB (master regulator of lysosome- and autophagy-related gene expression) and ZKSCAN3 (the transcriptional repressor of TFEB) regulatory cascades are perturbed with age remains unclear. In any case, upregulation of NRF2, TFEB, and FoxO activity has been associated with antiageing phenotypes and extended life spans (reviewed in [166]). The expression of several miRNAs that regulate autophagy is altered during physiological ageing. For instance, miR-34 is upregulated in *C. elegans* with age and inhibits the expression of the autophagy gene ATG9A in vitro [167]. Due to the conservation of miR-34 in different organisms, it is conceivable that such an effect also occurs in mammals.

### 4.3. CMA Decline in Ageing

Reduced CMA activity has been observed in aged human senescent fibroblasts and lysosomes isolated from old rats [137, 168]. Both substrate binding to the lysosomal membrane and transport into lysosomes decline with age due to a progressive age-related decrease in LAMP2A levels [168]. This is not due to reduced transcription of LAMP2A, but rather the result of (1) altered mobilisation of lysosomal luminal LAMP2A to the membrane upon activation of CMA and (2) replacement of its tightly regulated cleavage at the lysosomal membrane by a less regulated LAMP2A degradation in the lumen [169]. Although the cytosolic levels and activity of HSC70 remained unchanged with age, levels of lysHSC70 were increased in the oldest rats, which suggest an attempt to compensate for the reduced activity of the pathway with age [168]. The reduction of CMA activity with age probably contributes to the accumulation of oxidised proteins, which is characteristic of most tissues in old organisms. In fact, restoration of CMA through overexpression of an inducible exogenous copy of LAMP2A in the liver of aged rodents leads to reduced levels of oxidised and aggregated intracellular proteins [170].

### 4.4. Proteolytic System Crosstalk

Proteolytic systems (the ubiquitin-proteasome system and the various types of autophagy) are characterised by considerable crosstalk and the ability to compensate for each other. In fact, they share substrates, effectors, and even regulators. Blocking the proteasome can result in macroautophagy induction [171] and disruption of one type of autophagy can result in the activation of either the proteasome or different types of autophagy [120, 172]. It is thought that compensation between proteolytic systems may be sufficient for maintaining homeostasis under basal conditions, but not under (severe/chronic) stress conditions. In that context, it is interesting to note that dysregulation of the crosstalk between proteolytic systems with age may result in altered proteostasis. Indeed, proteasome inhibition has been shown to activate autophagy in young but not in old rats [173]. Schneider et al. found that, while other proteolytic systems compensate for CMA loss in young mice, these compensatory responses are unable to prevent proteotoxicity induced by stress (oxidative stress or lipid challenges) in old mice [174]. Either dysregulation in the insulin pathway (which may connect the proteasome and autophagy) or TFEB signalling (probably affecting different types of autophagy) with ageing may negatively impact on the crosstalk between proteolytic systems with age [173, 174].

## 5. Loss of Redoxtasis and Autophagy in Alzheimer's Disease

Ageing is the main risk factor for the development of a number of diseases, including neurodegenerative diseases, cardiovascular diseases, metabolic defects, and cancer. A clear example of the deleterious consequences of the already mentioned alterations in redoxtasis and autophagy with age is provided by neurodegenerative diseases, such as Alzheimer's disease (AD). AD, the most common form of dementia in the elderly, is a proteinopathy characterized by the accumulation of insoluble aggregates of amyloid *β* (A*β*) peptides along with other components in senile plaques, as well as the presence of neurofibrillary tangles of hyperphosphorylated tau. Overall, AD is considered a multifactorial process in which genetic and environmental factors along with increased susceptibility to stress with age influence each other, resulting in the loss of neuronal and brain homeostasis. As discussed below, both the loss of redoxtasis and autophagy may be part of a vicious circle with a crucial role in the pathogenesis of AD.

### 5.1. The Role of Oxidative Stress in AD

The central nervous system (CNS) is particularly vulnerable to ROS/RNS damage as a result of a high oxygen consumption rate, the abundance of lipids, and the reduced expression of antioxidant enzymes compared with other tissues [175]. Indeed, the “oxidative stress hypothesis” for AD and other neurodegenerative diseases supports that cumulative oxidative damage over time could account for the late-life onset and the slowly progressive nature of these disorders [176].

Many studies have shown increased markers of protein oxidation/nitration (such as protein carbonyls and 3-nitrotyrosine), oxidative-modified nucleic acids (as 8-OhdG), and AGEs in the brains of subjects with conditions ranging from mild cognitive impairment to advanced AD [176, 177]. However, whether oxidative stress is a primary cause or a consequence of some other event in AD remains elusive.

Different potential sources of ROS/RNS in AD have been proposed. Several lines of evidence indicate that A*β* itself can induce oxidative stress. For instance, the insertion of A*β* into membranes results in lipid peroxidation [177], while the effect of A*β* on microglial RAGEs produces proinflammatory signals and oxidative stress [178]. In a similar manner to A*β*, many studies support the hypothesis that modified forms of tau can produce ROS [179]. For example, mice overexpressing tau (P301S), a common mutant in tauopathies, show increased levels of carbonyls and a deregulation of antioxidant enzymes prior to neurofibrillary tangle formation [180]. Another source of ROS/RNS in AD may be damaged mitochondria. There is a general reduction in the activities of electron transport chain complexes in AD, which results in impaired mitochondrial respiration and defects in energy metabolism. Indeed, morphological, biochemical, and genetic abnormalities have been widely described in mitochondrion from AD patients [181]. A*β* was reported to accumulate in mitochondrial membranes, disrupting the electron transport chain and increasing ROS production [182].

Studies of antioxidant enzymes in AD have not shown consistent data. Aksenov et al. found increased levels of oxidative stress-handling enzymes in the parietal lobes, but not in the cerebella, of AD patients. The authors suggest that region-specific differences related to the magnitude of ROS-mediated injury are likely to contribute to variable neurodegeneration in different areas of the AD brain [183]. Another study showed elevated glutathione peroxidase, glutathione reductase, and catalase activity in specific brain regions in AD compared with normal control subjects [184]. We and others have observed increased levels of NRF2 protein together with upregulation of some of its targets, such as heme oxygenase 1 (HMOX1) and NADPH quinone oxidase 1 (NQO1), in the necropsies of AD patients [97]. Although there are contradictory observations that could reflect different stages of disease progression, these results support the notion of a compensatory antioxidant upregulation in AD brains.

### 5.2. Oxidative Modification of A*β* and Tau Proteins

Increased oxidative stress may affect A*β* and tau metabolism and function, leading to neurotoxicity. A*β* aggregation is accelerated by AGE-mediated cross-linking [185]. In fact, AGEs result in increased levels of A*β* per se through the upregulation of the amyloid precursor protein (APP), from which A*β* originates. Interestingly, this effect is abrogated by pretreatment with N-acetyl-cysteine (NAC), which points to its dependence on ROS [186]. Multiple studies have observed the oxidation of methionine in position 35 of A*β*. However, the functional impact of this modification is debatable, with some reports claiming it is critical to A*β*-induced oxidative stress and neurotoxicity [177] and others ascribing it a neuroprotective role [187]. The previously noted reduction in MSR with age may increase the quantities of A*β* and oxidised Met35 [177].

Tau is a natively unfolded protein that can undergo several posttranslational changes in addition to phosphorylation, including o-linked glycosylation, ubiquitination, SUMOylation, nitration, glycation, acetylation, and cross-linking [179]. The exact outcome and impact of these modifications on AD pathology remain largely unknown. It has been suggested that nitration favours tau oligomerisation and aggregation [188]. Moreover, prooxidant treatment of primary cortical rat neurons has been shown to significantly increase the aberrant hyperphosphorylation of tau in a GSK3*β*-dependent manner [189].

### 5.3. The Role of Defective Autophagy in AD

The oxidative modification and aggregation of A*β* and tau may be exacerbated by, and contribute to, impaired autophagic degradation activity. Autophagy impairment plays a crucial role in the pathogenesis of AD. In fact, excessive accumulation of autophagosomes and autophagic vacuoles (AVs) has been shown in the brains of AD patients [190]. This is likely due to incomplete autophagosome-lysosome fusion and digestion, possibly combined with induction of the initial steps of the autophagic process.

The accumulation of p62 and ubiquitinated proteins in the brains of AD patients has also been reported [191], indicating defective autophagy. Both APP and tau have been shown to colocalise with p62, suggesting their potential for being sequestered in autophagosomes for degradation [96, 192].

The underlying mechanisms for the defect in the clearance autophagosomes and their content in the neurons of AD patients are not yet fully elucidated, but several possible reasons have been reported. Familial forms of AD can be caused by inactivating mutations in presenilin 1 (PSEN1) and presenilin 2 (PSEN2). Presenilins are the catalytic subunits of the *γ*-secretase complex. Lee et al. suggested that full-length PSEN1 functions as a chaperone necessary for the glycosylation of the V0a1 subunit of the vacuolar (H+)-ATPase. This step is critical for its ER-to-lysosome transport. In the absence of PSEN1, the V0a1 subunit would fail to reach the lysosomes, impairing lysosomal acidification and, consequently, the proper function of this organelle [193]. However, a recent report revisited this issue, not finding lysosomal acidification impairment in cells lacking PSEN1 or PSEN2 [194]. Genetic studies have also identified several loci associated with AD risk, including the phosphatidylinositol-binding clathrin assembly protein (PICALM) [195]. This protein is implicated in the endocytosis of SNARE proteins, necessary for fusion of autophagosomes with lysosomes. Reduced function of PICALM has been reported in AD, based on the finding of reduced full-length and increased cleaved protein levels [196]. In fact, Moreau et al. showed modulation of autophagy-dependent tau clearance by PICALM [197]. Disorganisation of the microtubule cytoskeleton due to tau hyperphosphorylation can also prevent the transport of AVs to lysosomes, further aggravating this phenotype [198, 199].

Although AVs are a major reservoir of intracellular A*β* in the brain [200], the interplay between autophagy and A*β* is complex. On the one hand, A*β* may be degraded by autophagy, as autophagy induction has been shown to reduce its levels [201]. However, Yu et al. reported that autophagosomes may be sites of A*β* production, as they detected A*β* generation-related enzymes (such as PSEN1 and nicastrin) inside these compartments [200]. Indeed, autophagy impairment has been associated with reduced extracellular A*β* deposition and plaque formation, which would hypothetically result in more intracellular and possibly toxic accumulation of the peptide [96, 202]. Overall, these studies point to the existence of more than one deficit in the autophagy pathway in AD patients. This may favour the accumulation of not only aggregated proteins but also damaged organelles and lipofuscin, which, in turn, may result in increased oxidative stress.

Moreover, different A*β* and tau modifications may alter its autophagic clearance. Interestingly, a recent report by Caballero and coworkers showed a complex interplay between different mutations and posttranslational modifications of tau and selective forms of autophagy. For instance, the A152T tau mutation, associated with higher risk of AD, disrupts its degradation by endosomal microautophagy and is rerouted towards macroautophagy degradation. Moreover, a phosphorylation mimetic of tau in the microtubule-binding domain allows tau binding to lysosomes, but its translocation is disrupted. On the other hand, mimicking phosphorylation on the flanking domains results in impaired tau binding to lysosomes. Interestingly, cells expressing either of the different tau forms analysed in this study were unable to upregulate autophagic pathways in response to oxidative stress, which reduced cell viability [203]. Future studies may clarify the impact of different A*β* modifications on autophagic degradation.

Although one study observed decreased levels of BECLIN1 in AD cortex compared to control subjects [204], we and others have found upregulation of a number of autophagy-related genes in the brains of AD patients [96, 150]. These results are opposite to what is found in normal ageing—where decreased transcription of autophagy genes has been reported, pointing to a compensatory upregulation of autophagy. In the same line of evidence, we observed increased NRF2 and its target p62 in APP- and tau-expressing neurons in AD samples [96]. NRF2-deficiency has also been shown to result in increased oxidative stress and aggravated proteinopathy in different mouse models of AD [97, 205]. The lysosomal protease cathepsin D also accumulates in AD brains compared to age-matched nondemented control brains [206]. Altogether, these data may be interpreted as the unsuccessful attempt of the diseased brain to recover homeostasis.

## 6. Conclusions

While the ultimate causes of ageing are complex and multifaceted, knowledge of the cellular, biochemical, and genetic changes that accompany ageing continues to grow. There is strong correlative evidence that implicates the loss of redoxtasis and proteostasis in the process of ageing and disease development. Future research should provide a better understanding of the causal relationships between these processes, which will be crucial in prolonging life span and health span and in providing new powerful tools for the development of therapeutic approaches to a wide range of pathologies.
